# Endoscopic ultrasound-guided tissue acquisition of a focal liver lesion via the duodenum under fluoroscopic guidance

**DOI:** 10.1055/a-2686-7833

**Published:** 2025-09-04

**Authors:** Yuichi Takano, Naoki Tamai, Masataka Yamawaki, Jun Noda, Tetsushi Azami, Fumitaka Niiya, Masatsugu Nagahama

**Affiliations:** 1 Division of Gastroenterology, Department of Internal Medicine, Showa University Fujigaoka Hospital, Yokohama, Japan

In recent years, endoscopic ultrasound-guided tissue acquisition (EUS-TA) has become increasingly common for evaluating focal liver lesions 1 2 . However, accessing focal liver lesions from the duodenum remains technically challenging. Here, we report a case in which EUS-TA for a focal liver lesion was successfully performed via the duodenum by adjusting the orientation of the echoendoscope under fluoroscopic guidance.

The patient was a woman in her 60s. Abdominal contrast-enhanced magnetic resonance imaging revealed a 16-mm lesion in segment 4 of the liver ( Fig. 1 ), and EUS-TA was scheduled for diagnostic assessment.

**Fig. 1:**
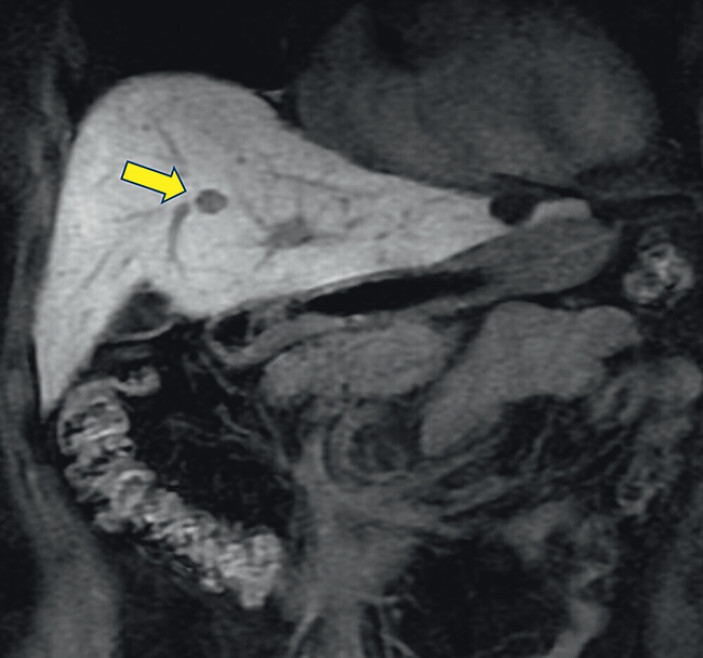
Abdominal contrast-enhanced magnetic resonance imaging revealed a 16-mm lesion in segment 4 of the liver (arrow).

A convex-array echoendoscope (GF-UCT260; Olympus Medical Systems, Tokyo, Japan) was advanced to the duodenal bulb. Initially, the probe was oriented caudally, preventing visualization of the liver ( Fig. 2 **a** ). Under fluoroscopic guidance, the probe was directed toward the liver ( Fig. 2 **b** ). The liver was clearly visualized, and the target lesion was successfully identified on the EUS image ( Fig. 3 ) EUS-TA was performed using a 22-gauge fine-needle biopsy needle (SonoTip TopGain; Medico’s Hirata, Tokyo, Japan). No procedure-related adverse events occurred ( Video 1 ).

**Fig. 2:**
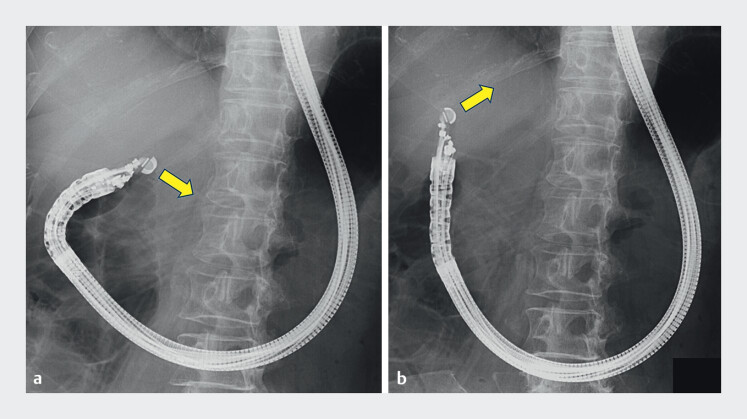
Fluoroscopic imaging showing the convex-array echoendoscope at the duodenal bulb. **a** Initially, the probe was oriented caudally, preventing visualization of the liver (arrow). **b** Under fluoroscopic guidance, the probe was directed toward the liver (arrow).

**Fig. 3:**
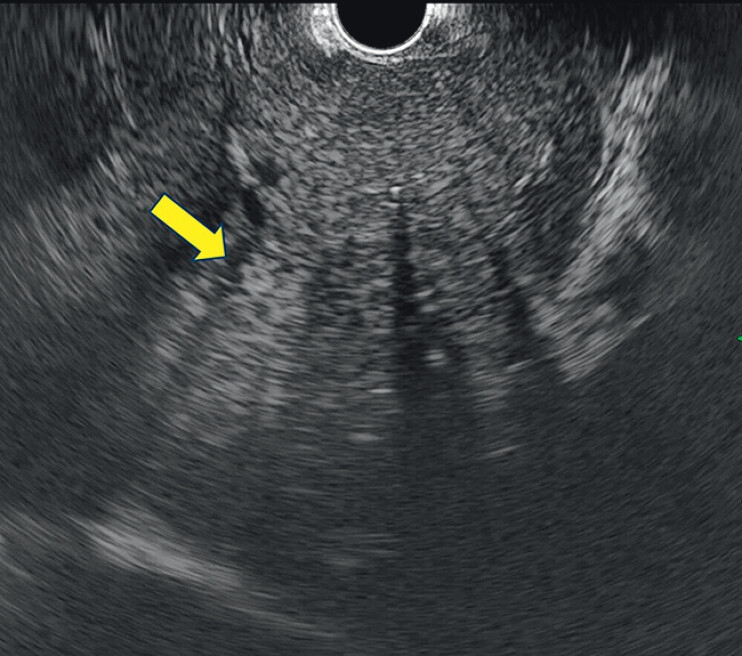
A hyperechoic lesion with a peripheral hypoechoic rim was detected in segment 4 of the liver (arrow). Endoscopic ultrasound-guided tissue acquisition was performed.

Endoscopic ultrasound-guided tissue acquisition from a focal liver lesion via the duodenum under fluoroscopic guidance.Video 1

Histopathological analysis confirmed adenocarcinoma ( Fig. 4 ). The patient was diagnosed with breast cancer liver metastasis and referred for systemic chemotherapy.

**Fig. 4:**
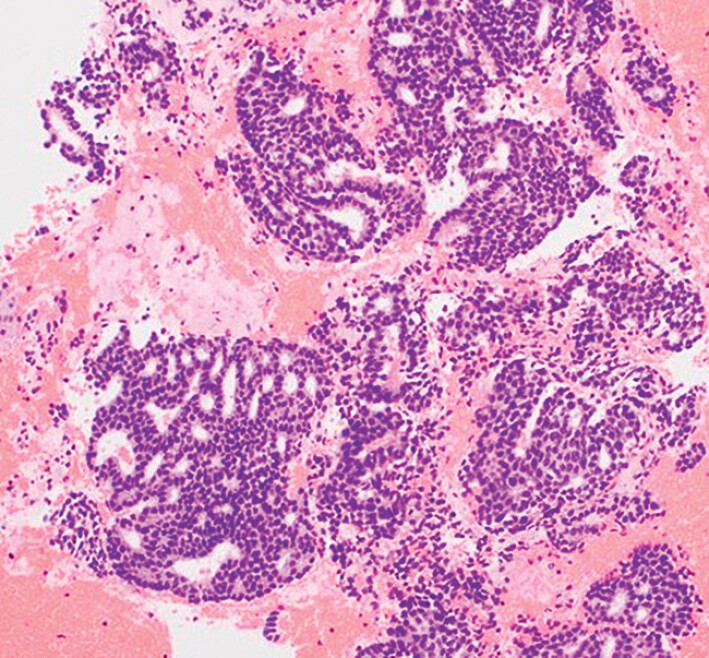
Histopathological analysis confirmed adenocarcinoma (hematoxylin and eosin staining, ×100). The patient was diagnosed with breast cancer liver metastasis and referred for systemic chemotherapy.

Visualization of focal liver lesions from the duodenum using EUS can be technically challenging. However, adjusting the orientation of the echoendoscope under fluoroscopic guidance allowed for precise lesion detection. This technique may be particularly useful for targeting focal liver lesions from the duodenal approach.

Endoscopy_UCTN_Code_TTT_1AS_2AF

## Notes

Endoscopy E-Videos https://eref.thieme.de/e-videos
*E-Videos* is an open access online section of the journal *Endoscopy* , reporting on interesting cases and new techniques in gastroenterological endoscopy. All papers include a high-quality video and are published with a Creative Commons CC-BY license. Endoscopy E-Videos qualify for HINARI discounts and waivers and eligibility is automatically checked during the submission process. We grant 100% waivers to articles whose corresponding authors are based in Group A countries and 50% waivers to those who are based in Group B countries as classified by Research4Life (see: https://www.research4life.org/access/eligibility/ ). This section has its own submission website at https://mc.manuscriptcentral.com/e-videos .
